# Perspective: Adhesion Mediated Signal Transduction in Bacterial Pathogens

**DOI:** 10.3390/pathogens5010023

**Published:** 2016-02-18

**Authors:** Sudha Moorthy, Julia Keklak, Eric A. Klein

**Affiliations:** 1 Biology Department, Rutgers University-Camden, 200 Federal St., Suite 520, Camden, NJ 08103, USA; smoorthy2404@gmail.com (S.M.); julia.keklak@rutgers.edu (J.K.); 2 Center for Computational and Integrative Biology, Rutgers University-Camden, 200 Federal St., Suite 520, Camden, NJ 08103, USA

**Keywords:** adhesion, signal transduction, virulence, uropathogenic *E. coli*, fimbriae

## Abstract

During the infection process, pathogenic bacteria undergo large-scale transcriptional changes to promote virulence and increase intrahost survival. While much of this reprogramming occurs in response to changes in chemical environment, such as nutrient availability and pH, there is increasing evidence that adhesion to host-tissue can also trigger signal transduction pathways resulting in differential gene expression. Determining the molecular mechanisms of adhesion-mediated signaling requires disentangling the contributions of chemical and mechanical stimuli. Here we highlight recent work demonstrating that surface attachment drives a transcriptional response in bacterial pathogens, including uropathogenic *Escherichia coli* (*E. coli*), and discuss the complexity of experimental design when dissecting the specific role of adhesion-mediated signaling during infection.

## 1. Introduction

Advances in transcriptomics have provided a wealth of information regarding changes in bacterial gene expression during infection. For example, uropathogenic *E. coli* (UPEC) adapt to intrahost survival by upregulating metal transport genes [1] and the oxidative stress response [2]. From a mechanistic perspective, it remains unclear what triggers this variety of transcriptional responses; the intrahost environment provides changes in oxygenation, nutrient availability, and presents a number of ligands for bacterial adhesion. In fact, recent studies have demonstrated that pathogens can sense attachment to host tissue resulting in changes in gene transcription. The notion that surface interaction can generate a physiological response in bacteria is not new. For example, in *E. coli*, it has been demonstrated that adhesion to abiotic surfaces can induce expression of envelope-stress genes via the Cpx two-component system [3]; however, the nature of the signal for adhesion-mediated Cpx activation remains unclear. Additionally, bacteria undergo a variety of developmental processes during biofilm formation [4,5,6]. Development of biofilms by *Vibrio cholerae* (*V. cholerae*) is a three-stage process. When planktonic cells first encounter a surface, they form transient interactions mediated by mannose-sensitive hemagglutinin [7]. Surface association results in the downregulation of flagellar genes and promotes the transition to a stably adherent biofilm. Transcriptome analysis shows that *V. cholerae* gene expression is differentially regulated in the various stages of biofilm development [5].

One of the major challenges in advancing the field of bacterial mechanical signaling is properly deconvolving the roles of chemical and mechanical environments in signal transduction during infection. In this perspectives article, we will examine the reports of adhesion signaling in UPEC; however, since data on mechanotransduction in UPEC is quite sparse, we will begin with an introduction to adhesion-signaling models in other pathogenic organisms.

## 2. Adhesion Mediates Physiological Responses in a Variety Of Bacterial Systems

**Figure 1:**
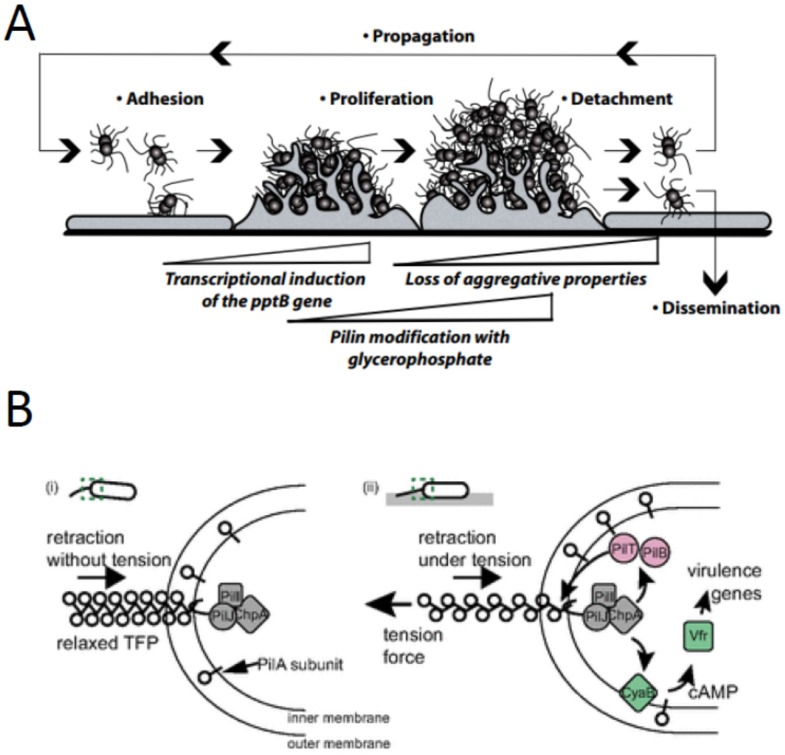

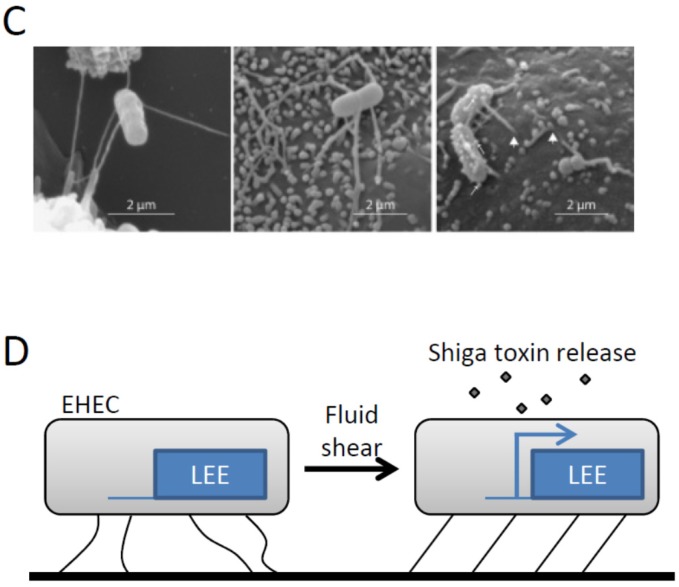
Adhesion regulates physiological responses in a variety of bacterial pathogens. (**a**) Adhesion of *Neisseria meningitidis* to host cells results in upregulation of *pptB*, posttranslational modification of pilin subunits, and dissemination of bacteria to enable colonization at distant sites. Figure reprinted with permission from *Science* [10]. (**b**) Type IV pili regulate adhesion-mediated signal transduction in *Pseudomonas aeruginosa*. One possible mechanism is that pilus retraction induces tension on the pili and changes the interaction between PilA and PilJ. Figure reprinted with permission from *PNAS* [13]. (**c**) Upon attachment to intestinal epithelial cells, enterotoxigenic *E. coli* exhibit broad changes in their gene expression profile leading to increased toxin production and changes in adhesion. One manifestation of the adherent transcriptional profile is the formation of surface blebs. Figure reprinted with permission from *Infection and Immunity* [15]. (**d**) Enterohemorrhagic *Escherichia coli* virulence factors, including Shiga toxins are, expressed from the locus of enterocyte effacement (LEE). Adhesion to HeLa cells induces LEE expression, which is further enhanced by fluid shear forces on par with those found in the intestinal tract.

In the context of host–pathogen interactions, adhesion of *Neisseria meningitidis* (*N. meningitidis*) to human epithelial or endothelial cells induces changes in the expression of ~350 genes [8,9], many of which have potential roles in virulence. After several rounds of division while attached to a host cell, *N. meningitidis* upregulates the phosphoglycerol transferase PptB, which transfers phosphoglycerol to the major pilin (PilE) in type IV pili [10]. This posttranslational modification disrupts inter-bacterial adhesion and allows individual cells to release and colonize new sites. It is hypothesized that the regulation of bacterial detachment may provide a selective advantage by enabling pathogens to avoid nutrient depletion and potentially to evade local immune surveillance [10] (Figure 1a).

The opportunistic pathogen *Pseudomonas aeruginosa* (*P. aeruginosa*) also relies on surface attachment to regulate virulence. *P. aeruginosa* can adhere to a wide variety of biotic and abiotic surfaces via type IV pili. A comparison between the pathogenicity of planktonic *versus* surface-associated cells revealed that adherent *P. aeruginosa* cells upregulated the transcription of a variety of virulence genes and were far more efficient in killing eukaryotic cells *in vitro* [11]. This adhesion-mediated virulence depends on the minor pilin PilY1, which shares homology to the mechanosensitive von Willebrand factor (VWF) domain [12]. The retraction of adherent type IV pili induces signal transduction through the Chp chemosensory pathway via interactions between PilA and PilJ [13]. Tension on type IV pili results in conformational changes [14]; thus, an intriguing hypothesis is that pilus tension regulates the interaction of PilA and PilJ to mediate a transcriptional response (Figure 1b).

Various strains of *E. coli* also demonstrate physiological responses to surface-attachment. Enterotoxigenic *E. coli* (ETEC) undergo large-scale transcriptional reprogramming upon attachment to intestinal epithelial cells *in vitro*; a number of virulence-associated pathways are induced including genes for toxin production, immunodominant peptides, and adhesion [15]. These transcriptional changes result in dynamic alteration of ETEC surface architecture, namely the formation of surface blebs and the upregulation of surface antigens including the adhesion molecule EaeH [15] (Figure 1c).

The virulence of enterohemorrhagic *E. coli* (EHEC) is highly dependent on type 3 secreted effector proteins, Shiga toxins, and adhesion factors (Tir/Intimin), which are encoded on a pathogenicity island termed the locus of enterocyte effacement (LEE) [16]. Transcription of the 5 LEE genes is induced by the LEE-encoded regulator (Ler) [17] which itself is positively regulated by GrlA [18]. Adhesion to HeLa cells induces LEE expression in a GrlA-dependent manner [19]. Furthermore, LEE expression is enhanced by fluid shear forces on par with those found in the intestinal tract [19] (Figure 1d).

## 3. Adhesion of Uropathogenic *E. coli*

Uropathogenic *E. coli* (UPEC) express several adhesive pili that bind to glycosylated host target proteins in the kidney and bladder and are required for infection. For example, type 1 pili bind to mannosylated proteins in the bladder, whereas P pili target a series of galabiose-ceramide moieties in the kidney. In cases of bladder infection, or cystitis, the primary virulence factor for UPEC is the type 1 pilus, a micron long filamentous surface structure consisting of repeating FimA subunits and a distal tip fibrillum (FimFGH). Pilus subunits are assembled and anchored in the outer membrane by a chaperone (FimC) usher (FimD) system via a donor-exchange mechanism [20,21,22]. The tip adhesin, FimH, binds to mannosylated uroplakin proteins found on the surface of bladder epithelial cells, accounting for its tropism to this organ [23,24]. Besides their role in initial adhesion, type 1 pili are also required during intracellular bacterial community (IBC) formation within epithelial cells, contributing to antibiotic resistance and evasion of the host immune response [25].

The mechanism of FimH attachment to host tissue provides an ingenious method for resisting washout due to recurring fluid shear stress during micturition. The 30 kDa FimH has two domains: a mannose-binding (lectin) domain and an anchoring (pilin) domain that anchors FimH to the fimbrial tip [26]. In the absence of tensile force, the pilin domain interacts with the lectin domain causing a twist in the β sandwich fold of the lectin domain. This loosens the mannose-binding pocket and leads to a low affinity state of FimH [26]. The application of tension across FimH separates the domains and allows the lectin domain to untwist and bind tightly to its ligand. This catch bond mechanism increases binding strength and bacterial adhesion under tensile force [27,28]. A similar mechanism for shear-enhanced adhesion has been reported for P pili attached to Gal-1,4-Gal via the tip protein PapG [29]. The ability to switch between high and low affinity binding states, as opposed to simply having constitutive high affinity, appears to be critical for proper pathogenicity. For example, the FimH G66R mutation results in increased binding to monomannose but loss of shear-enhanced affinity. Despite its overall higher affinity, this mutant does not result in long-term host colonization [30].

## 4. Adhesion-Mediated Bacterial Signaling in UPEC

From the host-cell perspective, adhesion of UPEC triggers a number of cell signaling events leading to endocytosis and internalization of the bacteria. FimH can bind to either α_3_ or β_1_ integrins [31,32] or uroplakin Ia (UPIa) [24,33] on bladder epithelial cells. Integrin binding results in actin remodeling via the activation of focal adhesion kinase, phosphatidylinositol 3-kinase, Rac1, Cdc42, and possibly Src-kinase [31,32,34]. In contrast, UPEC adhesion to UPIa triggers casein kinase II-mediated phosphorylation of the cytoplasmic tail of UPIIIa and increased intracellular calcium [35]. Inhibition of these processes results in decreased bacterial invasion.

Despite our understanding of host-pathways induced by bacterial adhesion, we know comparatively little regarding how UPEC respond to host-attachment. Early reports using differential display PCR found that P pilus adhesion upregulates *airS* expression [36] while type 1 pilus attachment inhibits capsular assembly by downregulating *kpsD* [37]. To our knowledge, the only published report of a global survey of type 1 pilus-mediated signaling comes from the Wishart lab [38]. In this paper, they attached a hyperpiliated K-12 strain (CSH50, *fimE1::IS1-*) [39] to mannose-agarose beads, collected RNA from attached and free cells, and performed transcriptional profiling by microarray. Their results showed an induction of protective metabolic pathways including formaldehyde degradation, assimilatory sulfate reduction, removal of reactive oxygen species, and removal of hydrophobic compounds. A number of these regulated genes are under the transcriptional control of the redox sensors OxyR and SoxS. Interestingly, *soxS* upregulation, along with its target genes, is associated with biofilm formation [40,41]. Furthermore, a number of other differentially expressed genes have been associated with biofilm formation, namely *ychF* [42], *ycfR* [43,44], *cysDNC*, and *cysJI* [42,45]. Thus, one could hypothesize that initial attachment to the host may prime cells for IBC formation.

**Figure 2:**
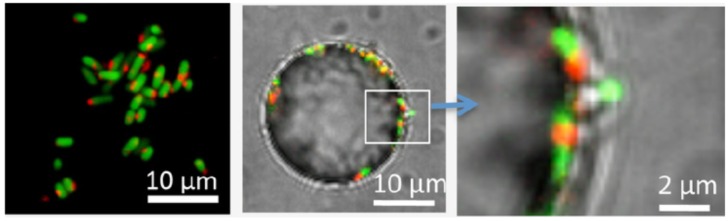
Adhesion-mediated signal transduction in *E. coli*. Adhesion of curli-expressing K-12 *E. coli* to polystyrene particles induces a surface-associated decrease in respiration. Cells constitutively expressing GFP were labeled with 5-cyano-2,3-ditolyl tetrazolium chloride (CTC) to monitor respiration (red stain). Shown are cells in suspension (**left**) or on the surface of a 25-µm particle (**middle** and **right**). Figure reprinted with permission from *PLOS One* [47].

An important caveat in interpreting these data is isolating the contribution of mechanical signals *versus* chemical stimuli such as local oxygen availability or chemical gradients. For instance, the adhesion assay in the Wishart report was performed in sealed tubes under rotation, where one would likely expect the environment to be fairly anaerobic. Oxygen availability may be of particular concern due to its role in regulating the switch between expression of type 1 and S pili [46]. Interestingly, adherent *E. coli* can be “tricked” into altering their metabolism regardless of the surrounding chemical environment. A recent study by Geng, *et al.* showed that surface-association decreases cellular respiration [47]. Curli-expressing K-12 cells were allowed to adhere to 3 µm polystyrene particles in solution; thus within the same system, comparisons could be made between adherent and free-floating cells. Using the fluorescent marker of bacterial respiration 5-cyano-2,3-ditolyl tetrazolium chloride (CTC), they found that within 10 minutes of adhesion, surface-associated cells had decreased respiration relative to free-floating cells (Figure 2). Since both cell populations were grown together in the same tube, they concluded that bacteria have a “sense of touch” which can be used to alter cellular physiology. In a similar vein, adherent *E. coli* can ignore local oxygen concentration to induce an SOS response to promote biofilm formation [48].

## 5. Future Outlook and Considerations

Since adhesion to the host is a key step for many bacterial pathogens, inhibiting initial attachment and/or downstream signaling pathways may be promising directions for the development of novel antimicrobial drugs. Indeed, there have been a number of publications demonstrating that mannose-analogues function as adhesion-antagonists for type 1 pilus binding and can prevent urinary tract infection by UPEC [49,50,51]. Additionally, pilus components are being used to develop antibacterial vaccines against organisms including group B streptococcus [52], UPEC [53,54], and *Enterococcus faecalis* [55]. The recent surge in publications investigating the role and mechanism of adhesion-mediated signal transduction in bacteria suggests that inhibition of these pathways may be a similarly attractive target for drug development. We eagerly anticipate the dissection of the molecular pathways regulating bacterial adhesion-signaling and the growth of a new paradigm in understanding host–pathogen interactions.
